# Time Orientation Technologies in Special Education †

**DOI:** 10.3390/s19112571

**Published:** 2019-06-06

**Authors:** Miguel Angel Guillomía, Jorge Luis Falcó, José Ignacio Artigas, Mercedes García-Camino

**Affiliations:** Deparment Ingeniería Electrónica y Comunicaciones, Universidad de Zaragoza, María de Luna 1, 50018 Zaragoza, Spain; 601084@unizar.es (M.A.G.); jiartigas@unizar.es (J.I.A.); garciacamino.mercedes@gmail.com (M.G.-C.)

**Keywords:** ambient assisted living (AAL), time orientation, technical aid, special education, accessible interfaces, assistive technology, collaborative design, personal autonomy, services provision, human/system interaction

## Abstract

A device to train children in time orientation has been designed, developed and evaluated. It is framed within a long-term cooperation action between university and special education school. It uses a specific cognitive accessible time display: Time left in the day is represented by a row of luminous elements initially on. Time passing is represented by turning off sequentially and gradually each luminous element every 15 min. Agenda is displayed relating time to tasks with standard pictograms for further accessibility. Notifications of tasks-to-come both for management support and anticipation to changes uses visual and auditory information. Agenda can be described in an Alternative and Augmentative Communication pictogram language already used by children, supporting individual and class activities on agenda. Validation has been performed with 16 children in 12 classrooms of four special education schools. Methodology for evaluation compares both prior and posterior assessments which are based in the International Classification of Functioning, Disability and Health (ICF) from the World Health Organization (WHO), together with observation registers. Results show consistent improvement in performances related with time orientation.

## 1. Introduction

### 1.1. Aims and Scope

Temporal orientation has been prioritized as a basic capacity to improve personal autonomy by education professionals of Alborada Special Education School in Zaragoza [1]. There has been a maintained collaboration between the University of Zaragoza and Alborada Special Education School in societal awareness of disability and in looking for solutions to children difficulties for more than 10 years. One collaboration area is time orientation (TO).

In such frame, we seek deeper understanding of training time-cognitive functions as well as a practical tool to improve this area in children with special needs. In order to assess and formalize intervention results, we have performed a classical empirical research methodology with pre-intervention assessment, intervention and post-intervention assessment. Goal population are children in special education schools. 

Goals of training with children include: (1) time perception capacity; (2) time and agenda management and (3) make task-changes emotionally softer by supporting awareness of their closeness. In this first validation process we structure a test for the two first objectives focusing in how this training affects to overall children performance that include time orientation capacities. We have selected items from ICF classification as we explain in methodology and have evaluated previous and posterior to the intervention. We have installed 12 devices in 12 classrooms of four special education schools in Zaragoza, and tested the changes in performance of 16 pupils.

### 1.2. Previous Works and State of the Art

#### 1.2.1. Human Time Orientation Capacities

The World Health Organization established in 2001 a taxonomy which details human functions: International Classification of Functioning, Disability and Health (ICF). Classification by the World Health Organization is a key document in working with human cognitive functions [2]. It sets human time orientation both as body functions and as participation functions. As mental body functions it is included in items b1140 “awareness of day, date, month and year”; as part of superior cognitive functions it is included in item b1642 “time management by ordering events in a time sequence” and b1802 “subjective experience of duration and passing of time”. As participation functions it is considered in the item 2306 “adapting to time demands”.

Another key document in time orientation concept is the PhD thesis of Janeslätt [3,4] which further details those items in a gradual cognitive evolution sequence: starting with time perception, it progresses to TO, further to time management and still further to global time processing ability. That work developed a time orientation assessment tool based on such concepts, KaTid, which we have found suited for an intervention as ours, although with higher cognitive level than our children have.

#### 1.2.2. Importance of Time Orientation Intervention

Time orientation may be seen as a skill in itself and an expression of other human capacities. Such human capacities end up building time orientation skill as an expression of more basic capacities, so it is expected that training improves such underlying capacities. In fact, our experience making time orientation tests with special education children shows their basic skill is far below expected, which in turn means they have a large adaptation and imitation capacity.

In this sense, it is useful to review some literature to show the large expected impact of time orientation training on personal autonomy, much further beyond the importance of managing the own time of day.

Although our aims are very concrete and practical, through training we expect to improve both specific autonomy regarding time and also basic cognitive functions as described below, including perspective towards future, reaching goals, risk awareness, anticipation to changes: basically making the child more autonomous and happier. Somehow widening the awareness of future time perspective may help children to acquire new perspectives, intentions, goals. It somehow means to widen the cognitive awareness, as following works indicate.

TO impairment is directly related to cognitive impairment, as described by the significance of temporal orientation tests to discriminate dementia [5]. TO is related to daily task management, so it can be used as an important measure of daily efficacy, as shown in [6]. Authors make an interesting detail of individual time orientations related to different life aspects: economic time, unorganized time, orientation toward the past, orientation toward the future, mastery of time, usefulness of time and time anxiety. Their paper also concludes culture plays an important role in time orientation and increasing awareness of future time makes people more adaptable to life changes and challenges.

TO capacity and tendencies build up specific personal time perspectives, which are related to having and pursuing vital goals [7]. Authors show how several styles of TO are related with styles of vital goals, finding the adaptive style has most positive impact. In this work they show how clear TO styles appear when correlating individual awareness of major goals in life interacting with age and gender.

Getting closer to the practical side, TO is basic for several types of learning, self-regulated, autonomous etc.: TO is needed for university learning online and virtual campus university studies [8]. For regular students, [9] shows good temporal orientation skills are related to better self-regulation and higher educational attainment.

Another important concept is “time perspective”: which may include past, present and future, influencing behavior, decisions and efficacy in achieving goals in life. Acquiring time orientation skills towards future, in normalized subjects, favors the display of a range of sustainable attitudes and behaviors, as detailed in [10]. Time perspective is related to behavior patterns and training is proven to modify such patterns: a recent study of the university Autónoma de Barcelona [11] explains how this perspective was used successfully in adolescence to reduce alcohol consumption. Basic mechanism is to train temporal perspective, which relates to risk perception and behavior (consumption). The study concludes that training in future time perspective does increase risk perception and modifies behavior positively. Extrapolating results with our children, time perspective acquisition will probably improve anticipation, lower anxiety related to changes and soften behaviors.

An interesting consideration about the importance of time perspective is found in [12] which relates mind orientation–inwards or outwards–with it, so that present time perspective increases inward awareness and past and future increases outwards. Many special education children need to connect to external activities to improve their daily autonomy. Extrapolating their results we could expect training in time orientation in future and present perspectives will train in parallel the connection with the outer world of children.

Future time orientation also has a positive relationship with goals approach and external regulation strategies [13], as proven by analyzing the results of different tests for each participant to measure those three dimensions.

#### 1.2.3. Other Works in Time Orientation Intervention

The seed-idea from which our time orientation intervention originates, comes from Svensk’s report about the use of a quarter of an hour as an understandable time unit and about sequential turning off of luminous elements in a row as a comprehensible representation of time passing [14,15,16]. He reported the use of this approach for phone assistance to cognitive disabled adults for their time management, as shown in [15]. The quarter hour clock [17] was derived from his experience, which is a reference in assistance devices and is being extensively used in AAL field. 

Many devices and specially software has been developed for normalized people, especially under agenda or productivity themes. Focusing on those specific for people with special needs, LoPresti in 2004 [18] stated time support for cognitive rehabilitation needs to include both time orientation (moment in time) and tasks associated.

Other few studies report good results by using time aids: in [19] authors showed that independence and autonomy should be considered as two separate concepts and report the importance of having frequent communication with the user to understand the usability of the aids. Another study [20] concludes the effectiveness of time aids to improve time-processing ability and managing one’s time in children with intellectual and developmental disabilities.

Most developments are conceived as a technical aid, not so many as an ambient intelligence tool for support in time orientation to people with memory impairments or attention deficit:

The quarter hour watch [21] represents events with pictures and the time remaining to the event shown as lighted points or dots (one point represents one quarter of an hour). It is also used to inform about the estimated duration of a task, based in “dot concept”. It has made a great benefit to technical aids community in which many adopted the dot as a standard.

Some focus in time intervals, as TimeTimer [22], which shows elapsed time in a close-to-analog way; Others focus on reminding the subject of tasks to come through the day, as WatchMinder [23] and TimeCue [24]. WatchMinder allows the user to create discreet cues throughout the day to perform specific tasks or modify or reinforce specific behaviors. TimeCue beeps or silently vibrates to discreetly signal tasks with a pictogram to describe the referred action.

An important development, pioneer in its time, is ISAAC [25,26], conceived as a cognitive prosthesis with personalized and procedural information. ISAAC is a patented, fully individualized cognitive prosthetic system which allows for the organization and delivery of individualized prompts together with personalized information.

Our work innovates in several areas, both as an individual time aid and as a special education class tool. As individual aid, it was born from the need teachers highlighted. Even when our device can be virtualized on a screen, teachers insisted that physical elements were important for many children as they did not have the same abstract capacity to understand different shape and color areas on a flat surface (screen). Moreover it is integrated in an ambient assisted living approach, so other available information can be used to further assist children. It also has different interfaces so it can be configured and programmed easily by a teacher with their own mindset for their class and also programmed in TICO-like [27,28] format so providing for individual and class activities related with time and agenda. Considering individual or group use, TOD is designed for individual training inside a group, being useful for the group and keeping in mind the social scenario where training occurs. TOD is integrated in an AAL platform for educational purposes together with other specific and interrelated services.

As far as software for schedule management, there was a boom of apps for time management, although few for people with special needs: “Picture Planner” [29,30] is an icon-driven software which helps users with cognitive disabilities and caregivers to construct and manage activity schedules. It is thought of for individual use. 

The “Student occupational time line”™ [31] provides both a text and visual overview of daily student-on-task school performance. Although it is designed for school use, its worksheets are printed with just text and colors for individual use. It focusses on sharing information about tasks and events for a specific child among those professionals in charge of him/her, with the intention to form a foundation for discerning personal, contextual, and temporal events that may contribute to dysfunctional behaviors of students with Asperger’s syndrome, autism, or sensory processing disorder.

Some more software developed for reminding and several styles of to-do-lists including step-by-step chunks that build tasks can be found in [32,33]. It is worth highlighting Choiceworks calendar [33] for it uses a similar strategy as we came up with to build up the agenda with pictograms.

None of the precedents fulfill the teachers’ needs: to schedule both individual and class group activities at one time, to call attention with time enough to avoid anxiety, to use a temporal unit easy to assimilate, to display the next event information in a friendly way, to be able to play recorded sound as mum’s voice, to configure it in the same fashion teacher manage class agenda, to integrate it in an AAL platform and to use TICO as input interface to be cognitively coherent with the rest of developments for those children. Intervention has further developed into different areas, as time orientation, memory support, anticipated notification of events progressing and changes, and time-planning or agenda-planning training.

In our previous studies [34,35], tasks description images were used for the various activities (in school, day center and at home) together with the row of luminous elements. The joint use of the foregoing caused the perception of time to show a substantial improvement. Further technological functions the prototype has achieved are reported in [36,37]. Next section summarizes the key technological aspects of the time orientation device validated in this work.

## 2. Time Orientation Technology Description

### 2.1. System Evolution

Our Time Orientation Device (TOD) uses the idea of a “dot” as a time unit. This idea is frequently used in special education and also in supporting to cognitive impaired adults. TOD was first designed for people with Alzheimer’s disease, for their relatively long phases in which the person is able to perform his/her daily tasks only if reminded.

**Figure 1:**
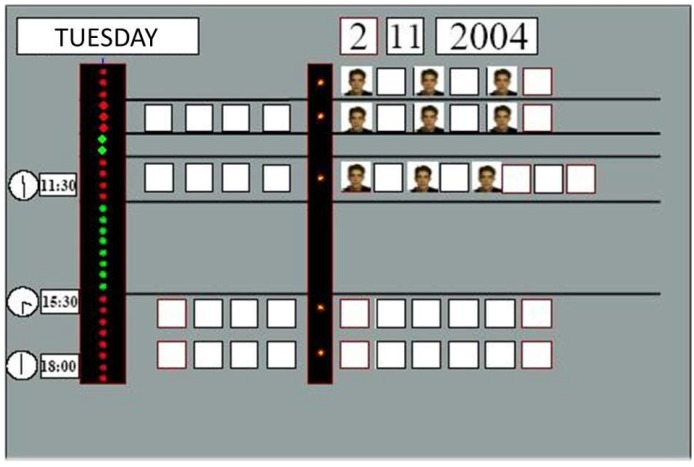
First adaptation of TOD to blackboard agenda.

When re-designed for special education school we kept the essence of a vertical “dot time display” adding display areas for class activities, taking as template their blackboard agenda. In a previous version and for activities related with one child going out from classroom, a second vertical column was added to anticipate children changes and give them an orientation in events to come, their duration and time left for them. By default, each element of the vertical line, a dot, represents 15 minutes. This first version is shown in Figure 1: time row on the left is for activities that are common to a class; right side column is for individual events, as a pupil going to physiotherapy or to the gym.

**Figure 2:**
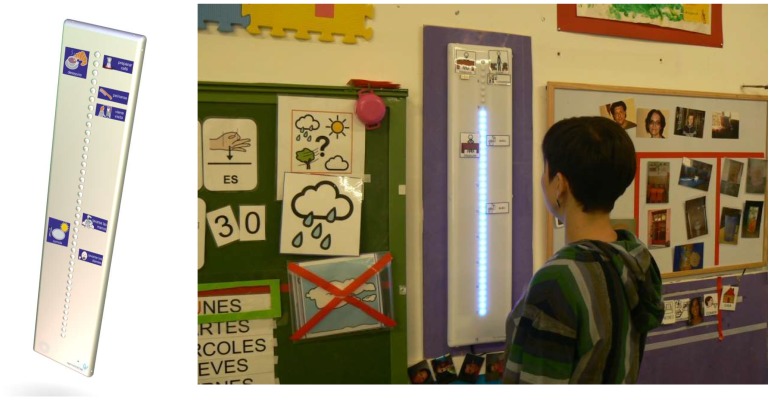
Second version and an example of use in the classroom.

The second version attended to both the flexibility of setting and use improvement and manufacturing, normative and homologation needs: the row of time dots become a closed box as shown in Figure 2, which then could be attached to the blackboard agenda, becoming much similar to its original version for elderly, which was hung in the living room or kitchen.

Current TOD adds a dedicated small computer attached to the time display with a small screen for pictograms and a loudspeaker for multimedia information. User software has been improved giving the teacher a wide configuration range.

The current system is composed of:

- A physical display in a long white plastic box with a row of several (40) luminous elements, with area at both sides to attach pictograms glued to magnets;
- A single-board computer which is attached to the display, in which the software runs;
- A speaker for melody or voice messages associated;
- A screen to show the pictogram of the current action or a simple pictogram sequence;
- The software package to program the agenda and configure played and displayed information.

### 2.2. Prototype Description

**Figure 3:**
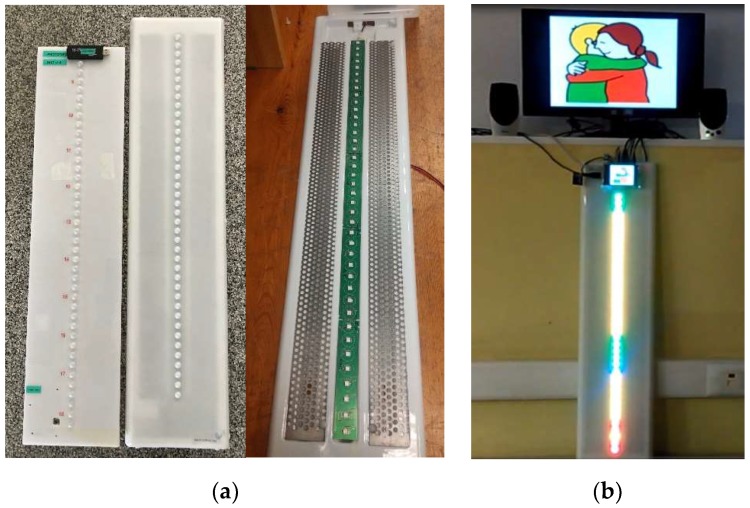
(**a**) TOD version 2: outside aspect and inside distribution; (**b**) TOD version 3: outside aspect.

Main element of the display is a row of luminous elements, (RGB LED’s) fitted in a molded translucent plastic panel of 81 × 19 × 6 cm. Such elements are configurable both in color and brightness from the software (Figure 3 and Figure 6). The row of luminous elements is set in vertical. To both sides there is a metallic panel inside the plastic which allows for magnets with time or tasks indications to be easily attached (Figure 3a).

Each element represents a time unit. Cognitive studies from Svensk [14] concluded a quarter of an hour as most adequate time unit for each element for global cognitive disabilities, so that is the default value set. Flexibility was demanded to this regard: some elder residences needed their agendas divided in units of half an hour and some extra functions as behavior contention needed only minutes, so time is configurable from the software application.

By default, LED color represents the moment in which the task is, this is past, current or future: each LED either gets off or changes color with the passing of time. Anyhow, color can be decided and set also with full flexibility from the software application.

Events which are to come soon are announced in a visual and acoustic way by the dedicated computer located in the upper part of the TOD. So stimuli to draw attention are given with sufficient time to avoid anxiety and to motivate the user.

#### 2.2.1. Electronics

Electronics consist of the specific TOD display which is a hardware controlled by I2C communication and a dedicated computer for configuration, human machine interface (HMI), network communications and other software modules which integrate the TOD in the framework of an AAL platform for education.

Display electronics are five LED boards which communicate power supply and data lines sequentially. Each one includes eight high-brightness RGB LEDs together with its control chips (MAX7315). Every board has three control chips, one for each basic color, and is managed by an I2C interface. All LED boards are connected in series to be managed by a single I2C port.

**Figure 4:**
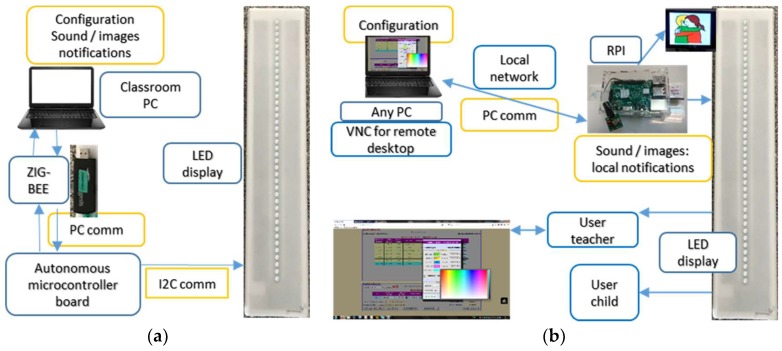
(**a**) TOD version 2; (**b**) TOD version 3.

Current prototype uses a Raspberry Pi (RPI) as a dedicated computer: it is a low cost single board computer specifically developed for educational purposes. Previous versions used a Zigbee microcontroller module with a Zigbee communication circuit to operate the LED boards from a remote computer. The autonomous microcontroller board provided autonomy from the classroom computer, however limiting functionality about multimedia feedback and connectivity (Figure 4a). Current version boosts its connectivity, multimedia feedback and database management capacities (Figure 4b) by being equipped with a RPI.

The current modular system shown in Figure 4b can be accessed either via remote desktop from a smart terminal/computer or as a http page, so accessibility is easy from different hardware platforms. From the second to the third version, intelligence for the device migrated from the microcontroller board to which needed communication with the classroom PC, to the RPI dedicated computer which fully controls the device, LEDs, images and sound communications. Suggested by teachers, also from second to third prototype we have added a 3.5″ 320 × 480 TFT screen to display the pictogram associated with each of the scheduled tasks, a set of speakers to reproduce locally the sound associated with corresponding task, a Wi-Fi dongle for wireless communication and a webcam. LED boards are controlled through the RPI- GPIO connector emulating I2C communication standard.

#### 2.2.2. Software

TOD uses Raspbian Jessie operating system, which is a free and open source operating system optimized for the RPI hardware.

**Figure 5:**
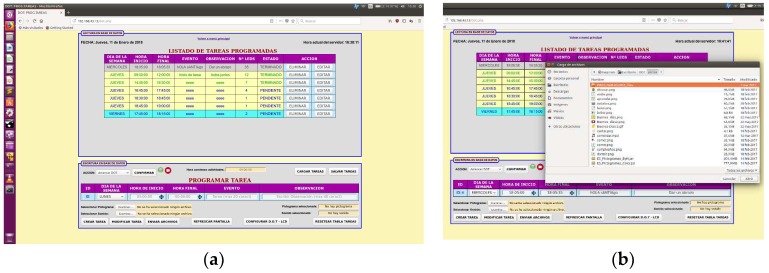
(**a**) Spanish TOD HMI main menu; (**b**) pop up window for multimedia file selection.

**Figure 6:**
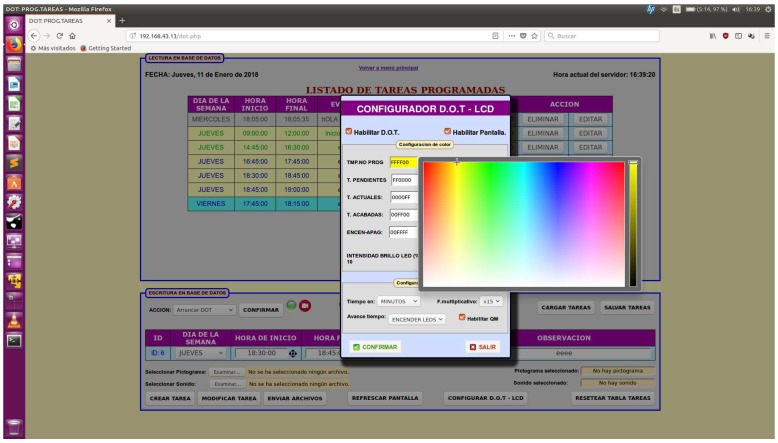
TOD color and brightness - with test option - and time configuration window.

Software includes a configuration module shown in Figure 5 and Figure 6, an open source HTTP web server (Apache) as well as a general programming language of server-side code for dynamic web content development (php), a database data management system (MySQL) and a tool to manage MySQL databases (phpMyAdmin). Such architecture provides for solid user access management and easier integration in the AAL platform with other applications by sharing information through the database.

Multiple web applications have been developed to handle several databases (users and permissions, TOD configuration and logs among others); besides, software also runs some Python applications for the management of webcam, I2C communication, video and audio playback applications and future external sensors and actuators via RF433 devices.

#### 2.2.3. Communications

Communication with the system can be done through the connection - as a client - of an SSH terminal and as remote desktop, either locally (connecting directly to the RPI via Ethernet or Wi-Fi) or remotely since the system is provided with a DDNS service. This allows modifying the TOD tasks by programming them remotely. In order to improve the usability and flexibility of the system, communication can be established from any computer, smartphone, tablet, regardless of the operating system used (Windows, Linux, Android, Mac OS, etc.).

#### 2.2.4. User Interface Description

User interface integrates feedback obtained by several specific workshops held with teachers and previous trials. As developer’s strategy we have tried to maintain it simple for basic and standard configurations through default values and options to edit a configured registered setup and save it as a different one. At the same time, from special education school, full flexibility was demanded, so system also allows providing full flexibility for all foreseen intervention actions through more elaborated HMI functions.

When connecting to the TOD website, one can see the control environment shown in Figure 5. Menu screen is divided into two windows: scheduled task information on top and task editing on bottom area. 

Schedules task information window shows current date and time and the list of scheduled tasks for the whole day and the whole week, together with the selection tool to delete or edit tasks one by one. Task status in time (past, current and pending) is coded with different color patterns: background color regards days: gray for days gone by, yellow for tasks for the current day and cyan/blue for tasks for future days. Font color displays task time regarding present moment: finished tasks have green font, current tasks purple and pending tasks blue.

The bottom window in Figure 5a shows the editing window, where one can: (1) create new tasks; (2) edit any scheduled task to change the day of the week, start time, end time, name of the task and include notes; (3) send pictograms and sounds to a local gallery folder for later use; (4) associate a pictogram and a sound to a task (Figure 5b); (5) load/save tasks configured from/to an external file; (6) initialize the list of tasks; (7) display start time of daily activity; (8) turn on/off a webcam to assist user by visualization of the elapsed time or the environment.

Some pop-up windows for each task are shown in Figure 6 which allow for further editing: (9) enable and disable touch screen; (10) configure preferences in color codes and brightness of pending, current, finished, unscheduled tasks and set the tasks that turn on and off LEDs. It gives an option to perform LED color tests to directly check color outcome in the physical display; (11) configure time base (hours, minutes, seconds) and multiplicative factor for each luminous dot (×1, ×5, ×10, and ×15), having “minutes” and “×15” as default value (each dot means 15 min as default).

Configuration is saved in a MySQL database with access protection through username and password through the application phpMyAdmin. It can also export the contents of the table to a file.

## 3. Evaluation

### 3.1. Summary

The evaluation experience has taken place in four different special education schools. We installed 12 TODs for 4 months. Another two devices were installed in staff rooms for teachers to test. We gave user training courses to the teachers involved and became available for support via phone assistance and visiting. We also designed the selection of participants together with the school; designed the assessment tool based in the ICF; informed and made the consent forms for each child’s counselor; performed individual assessments and analyzed the data obtained. Study design has prioritized the direct effect in children’s skills and its use as an educational tool.

Teacher’s teams were trained in configuration and use of the device as well as in observation dynamics and its basis.

The evaluation took 16 ICF items to assess the effect of the TOD as an education tool, including items related to attention focusing, social interactions involved and tasks execution. It was performed by evaluators with solid training and experience in ICF assessment. It is globally concluded that TOD is a valid assistive instrument. There are evidences of skills improvement most possibly because of automatism generation and little because of learning, as expected in this limited time span.

### 3.2. Methodology Goals, Constraints and Overall Description

Methodology’s main objective is assessing the effects of the use of the TOD as educational tool on the children capacities or skills related to “activities and participation”, in the World Health Organization notation. Some influencing circumstances of our target population are the enormous heterogeneity in children’s capacities and the limited number of participants available with a set capacity range. As our sample is little and in order to get some significance with the results, a structured methodology is designed. In turn this implies the need to limit both children profiles and number and type of events to test.

Sequence of evaluation begins with an ICF assessment of the different items of using the TOD in class, after one week of training. TODs are used during the experimentation period and teachers register their observations. At the end of the experiment’s period, a second ICF assessment is performed: results will come from the comparison of the two assessments and the analysis of the observation registers.

The experiment is based on the notification and execution of two tasks per day for each child with a fixed action program in each of the four participant schools. For each task it consists of TOD first announcing the action with a visual stimulus and then with an auditory one: first changing the color of the element representing the moment in time we are at and showing a pictogram to describe the activity to come. Pictograms are taken from the ARASAAC suite [24], widely used at school and so familiar with most children. Auditory stimuli come later and are reserved to the executive moment promoting the action. This notification strategy has been taken from the standard consensus in risk prevention notifications where signals indicate actions and sound the moment to execute them.

For each child we programmed an individual event and a group event. This way both dimensions are assessed and both dynamics are included: the separation from the group and the mimicry in group events. Separation means the child needs to take the initiative and not follow the others, where difficulties or good-functioning are expected. An example of individual notification may be the action to go to speech therapy; group example may be going for lunch or brushing the teeth with the rest of the group.

### 3.3. Participants

#### 3.3.1. Selection Process

First user analysis came up with several types of users involved: it included children, teachers, parents of children who supervise and authorize their children’s participation and school direction boards. First step was given with direction boards and in some cases to the educative assembly in which parents, teachers and school staff are represented. Presentation included description of the tool, its main features, main objectives of this project, and guidelines of the assessment process. Teachers offered their participation as volunteers and they chose the children with which they would work. Then teachers explained to families invited to participate, having the informed consent signed and in deposit in the same school.

#### 3.3.2. Special Education Schools and Classes

Participant schools have been three public schools—Alborada, Piaget and Angel Rivière, and a private one, San Martín de Porres (from ATADES association) as shown in Table 1.

Assessment focuses on individual effect, so methodology chose to have a child per class as a starting point. Anyhow as the number of children was low, this was optionally increased to having two children per class, which was chosen in four classes, having another eight with only one child, so finally the structure was:

(1) Four classes with two children per TOD;
(2) Eight classes with one child per TOD;
(3) A total of 16 children have used TOD in their daily school routines, during 3.5 months.

#### 3.3.3. Participants Description

Following tables detail some features of participants as data provided by schools, from age span to disability. Table 2 shows the age span of participants was very wide, from 7 to 19 years old, to increase the number of selected participants.

Table 3 shows the high grade of disability most of our participants had.

**Figure 7:**
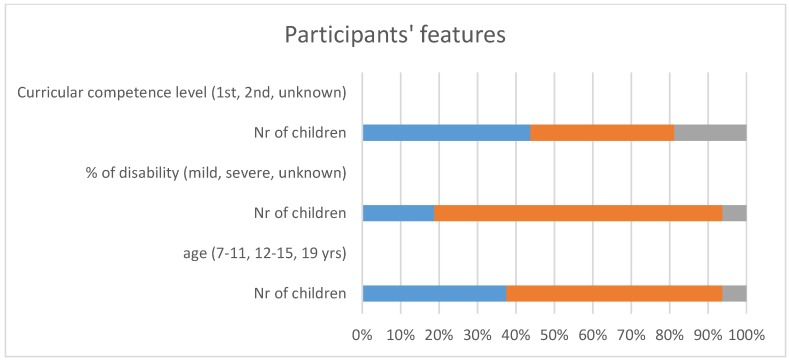
Participants’ features.

Children curricular competence level follows in Table 4:

Figure 7 summarizes the participants’ features. Most children present a high level of disability, ages ranging from 7 to 19 years old and with a low curricular competence level equivalent to 2 to 5 years of normalized development.

### 3.4. Evaluation Process

Evaluation process is structured in several phases, collaborating in each one with the school team. Sequence of actions is detailed as follows:

- Project presentation to school direction boards.
- Project presentation to teachers. Selection of candidates both children and teachers.
- Data retrieving from selected children. Translation into ICF.
- Training to teachers: group activity.
- Installation of TOD’s in the classrooms.
- Individual training and reinforcement, direct and telephone assistance.
- First ICF assessment of use of TOD in class.
- Use of TOD as planned, observations registered by teachers.
- Second ICF assessment of use of TOD in class.
- Teachers interviews regarding functionality and usability of device in the class.
- Debate group: group meeting in which incidences are put in common, solutions applied, opinions shared about TOD as educative resource, repercussion of use of TOD over human behavior.

Regarding the use in the class there was an agreement to turn the device on as soon as the class started and have it on during all school day. This way time passing was represented by sequentially turning off luminous elements and having color and sound notifications at the time configured. Teachers observe reactions from children and fill in a register template which was sent to researchers weekly.

#### 3.4.1. Manuals and Protocols

Together with direct training, several manuals were provided with the installation of the TOD:

Installation manual and process: This manual describes actions to have the system installed. Often teachers would accomplish installation themselves, so we checked future sustainability and manual understandability. In each school the situation was different: in Alborada and Piaget there were computers in the classrooms, in which a freezing process is used to avoid configuration to be changed by the user. In ATADES and Rivière there was no computer so we brought some that children didn’t use.

Location Manual: Location of TOD affects its efficacy and children perception. Recommendations for TOD location were developed, based in ergonomic and visual attention focus for children. It later included specific guidelines after visiting special education classrooms.

**Figure 8:**
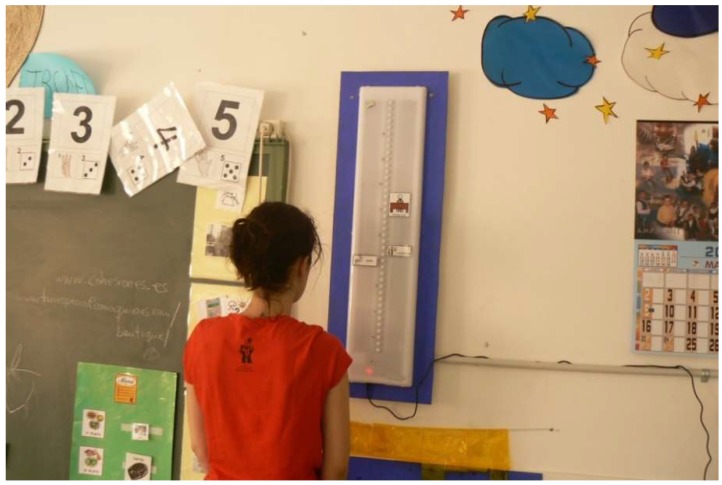
TOD without screen installed in a classroom, with lights off.

Figure 8 shows the amount of information surrounding the TOD, once located in a class: Summary of keys for location:

- Install in a space which is as free as possible of other stimuli so perception is easier and with less error margin.
- Situated in front or in diagonal of children’s place in the class, for visibility reasons.
- Locate it in a place with no direct artificial or sunlight to avoid reflected light which could make difficult its discrimination.
- The eye-height of children should be in the middle of the device.

User’s manual: It explains how to configure the events, notifications, LED color, etc.

Observation protocol: It explains the way in which teachers are expected to observe and act with the TOD experiment and which information to collect for validity of research action.

#### 3.4.2. Usage Incidences

Difficulties of observation and assessment of usage:

Volume of sound notifications was low when the environmental noise was high or other programs played videos or music.

Some devices had no enough space for a good visual discrimination due to the excess of information in many classrooms.

When school changes from split shift to continued day there were some difficulties in programming events, which was solved with a software update.

Other assessment difficulties were inherent to the type of children we work with. It was relatively frequent to have absences due to sickness and alterations of their mood due to changes in medication, anxiety crisis, crying, aggressiveness, etc.

On the other hand, schools try and do several excursions and activities out from the classrooms, having then leaved from the experiment. Still other factor was the work leave from some teachers.

### 3.5. Assessment Test Design, Based in ICF

We used a double assessment design, performing individual assessments before and after the experience, based in ICF. This tool has among its applications the capacity to assess the impact a support technology has on its users.

This assessment has some particular circumstances to take into account:

- Enormous variability of users and their behavior and difficulties which greatly depend on their level of well-being through the day.
- Short time scope available. Available action took 4-5 months with holidays in between, which is considered not enough to have stable modifications in changes and tendencies that some users presented.
- Selection process leaves experiment with few candidates, so we have a wide age range and a relatively small number of participants which only allows an approximation to individual results.

ICF assesses the functioning of people to give answer to demands in various areas of life. Related items are found in the chapter of activities and participation. This chapter makes reference to vital aspects related with functioning both from an individual and social perspective. All 16 items selected correspond to this section. They are considered in the following subsections:

(1) Learning and applying knowledge.
(2) General tasks and demands.
(3) Communication.
(4) Mobility.
(5) Self-care.
(6) Daily life.
(7) Interactions and interpersonal relations.
(8) Main areas in life.
(9) Community, social and civic life.

Methodology selects first items related with the activities involved in the task the TOD indicates. “Activity” is the actual making of a task or an action by an individual, what is done and how. “Limitations in the activity” are the difficulties a person may have in making some activities.

A set of 16 ICF items of activities and participation were selected for this assessment: those describing capacities that were implied in giving answers to demands related with learning time orientation. Selection then was made by relation with demands from TOD: look, listen, make a simple task, understand an icon, etc. Difficulty percentage is referred to them:

(1). Learning and applying knowledge:
  A. Sensory experiences with intention
    1- Look at: set the looking intentionally towards the interface.
    2- Listen: sound perception and set the looking towards the source
  B. Basic learning:
    3- Basic skills acquisition: to become capable of executing indicated actions.
  C. Knowledge application:
    4- Centering attention: Focus intentionally the attention on stimuli while they happen.
    5- Solve simple problems: To take exploratory actions and/or find solutions to achieve an objective.
(2). General tasks and demands:
    6- To carry out a simple task: Execute a task after receiving the notification.
    7- Complete daily routines: establish and fix time patterns and sequences from TOD notifications.
    8- Stress management: keep a behavior inside tolerable margins during TOD process
(3). Communication:
  A. Communication—reception
    9- Spoken messages communication-reception: understand teacher’s comments and from others if they were.
    10- Symbols and signals communication-reception: Understand the meaning of symbols and auditory stimuli.
  B. Communication—production
    11- Speak: in an understandable way with communicative intention.
    12- Non-verbal messages production: using signs, drawings or any other non-verbal messages to express him/herself and/or communicate something.
(4). Mobility:
    13- Walking short distances. Walk indoors with an aim.
(7). Interactions and interpersonal relations:
  B. Particular interpersonal relations:
    14- Relate with strangers: Establishing temporal links with strangers with specific purposes (in relation with evaluators).
    15- Relate with people in authority position: keep a respect relationship with professionals
    16- Informal relations with peers: with respect and affection

For each item, a difficulty percentage is defined as shown in Table 5:

## 4. Results and Discussion

### 4.1. ICF Assessment

From the 16 children involved in the training, finally 10 had enough data to provide a valid assessment with before and after experience test. In order to avoid any misinterpretation of the underlying mechanisms involved in getting the information and performing the indicated actions, we count the number of items that children perform with less difficulty in the second assessment as compared with the first one. We plan next evaluation actions that will seek insights into subjacent mechanisms by adding more observation registers and having longer training times. Moreover, longer training times are needed to infer learning results.

All of them show improvement both in qualitative and quantitative considerations. For a global quantitative measure of such improvement we introduce a new parameter “level of restriction”, which measures difficulty percentage in the overall test. Each difficulty level is considered in a range of % of difficulty as Table 5 shows, together with the mean value of such % per interval:

### 4.2. Individual Data and Results:

#### 4.2.1. ICF Assessment

Data in Table 6 shows the first ICF assessment of 16 selected items for child code 010201. Twelve of the items fall in category “large difficulty” and four in “moderate difficulty:

The main evaluation parameter to infer improvement is “level of restriction”: It is the mean value of the difficulty level % per restricted items:

(1) $$Level\;of\;restriction=\frac{\sum_{difficulty\;levels}[\left(\mathrm{nr}\;\mathrm{of}\;\mathrm{items}\;\mathrm{in}\;\mathrm{difficulty}\;\mathrm{level}\right)\times \left(\mathrm{dificulty}\;\mathrm{mean}\;\mathrm{value}\right)]}{\sum_{difficulty\;levels}\left(\mathrm{nr}\;\mathrm{of}\;\mathrm{items}\;\mathrm{in}\;\mathrm{difficulty}\;\mathrm{level}\right)}$$

Moderate difficulty has a medium percentage of 37% and large difficulty 72.5% (Table 5), so for this child’s first assessment our level of restriction is:

(2) $$Level\;of\;restriction=\frac{\left(4\times 37\%\right)+\left(12\times 72.5\%\right)}{16}=63.63\%$$

The second evaluation figures are shown in Table 7. In the example for child code 010201, five items assessed as large difficulty in first evaluation moved to moderate and light difficulty in the second, so increasing three overall in moderate and two in light. This results in a decrease of the level of restriction to around 50%, improving it by almost 14%.

#### 4.2.2. Observation Templates for Teachers

**Figure 9:**
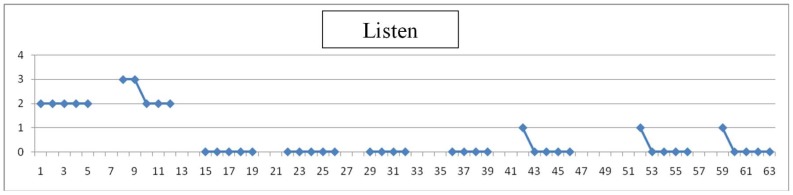
“Listen” reached soon a level of “no difficulty” with training.

**Figure 10:**
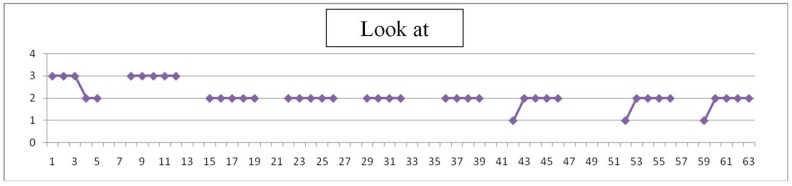
“Look at” reached soon a level of “light difficulty” with training.

**Figure 11:**
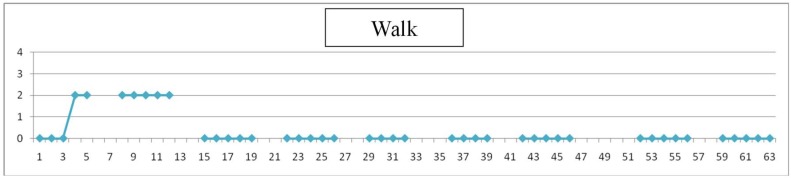
“Walking” reached soon a level of “no difficulty” with training.

**Figure 12:**
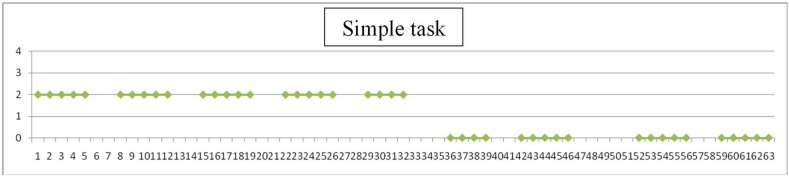
“Simple task” reached a level of “no difficulty” at midpoint of training period.

Each day teachers have assessed the degree of difficulty of simple items (values 1 to 4), as listen, look, walk and make a simple task. An example is shown in Figure 9, Figure 10, Figure 11 and Figure 12, where horizontal axis represents days of training for evaluation experience. Gaps in data correspond to weekends.

Assessment is here subjective and very personalized. Teachers know the children for months or years and have built a relation with them through time: on one side, this makes them more suitable to detect changes from their common pattern, even subtle changes or changes that are not focused in the evaluation designed; on the other side, observation may be altered due to affection and projected expectations towards children. Significance of the observations is very valuable to modulate and complete the perspective of individual results. An example follows in Figure 9, Figure 10, Figure 11 and Figure 12 of the type of data acquired and some open interpretation. A lower value in those figures means greater autonomy level: 0 means no difficulty for that activity and 4 means total difficulty, according to the five levels defined in Table 5. An example of this evaluation for one child is shown in Figure 9, Figure 10, Figure 11 and Figure 12.

Listen: This child has improved the function “listen” through the days. In this context, listen means an intention. As shown in Figure 9, data shows it reaches a non-difficulty level in two weeks time. It also shows that on Mondays difficulties are larger, while from Tuesday to Friday they improve systematically.

Look at: “Look at” means an intention. Improvement is reached also in two weeks time, although difficulty doesn’t come down to zero.

Walking: Walking is not just wandering, but needs the intention to go to a specific place, with a goal. In this case child has had no problem in walking, after a two weeks period of training.

Perform a simple task: This task has taken longer to show any improvement. At midpoint in the process it improves from “moderate difficulty” to almost “no difficulty”.

### 4.3. Overall Results

**Figure 13:**
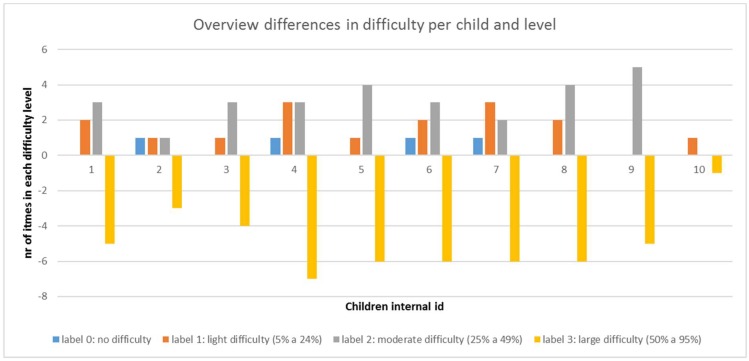
Overview of differences in number of items per difficulty level for the 10 children assessed.

From ICF assessment we obtain a “difference” vector, defined to register the differences between first and second assessments. Its elements are the subtractions of the number of items that fall in each difficulty level as assessed after the training and prior to it. A graphic representation of differences in number of items per child and difficulty level is shown in Figure 13. Total difficulty is not shown as there has been zero items in such category in all cases. Negative values of the number of items in “large difficulty” level means they have disappeared from that category and so fall into less difficulty ones. This graphic shows improvement is consistent for every single child.

**Figure 14:**
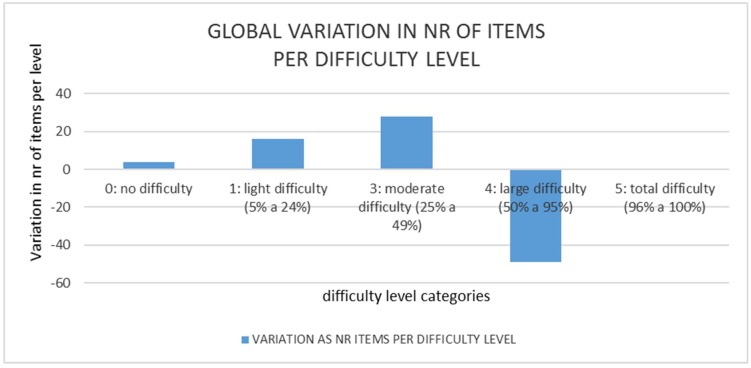
Number of items (49) that had large difficulty and improved into less difficulty categories.

We define “global improvement” to measure the overall result by adding up all item contributions per difficulty parameter for each individual child. As shown in Figure 14, 49 items did decrease their difficulty level from “large difficulty” into lower difficulty categories: this is an improvement in around 30% of the items.

Studying the group as a whole experiment, Figure 14 shows the global difference vector (adding individual ones) showing up graphically the decrease in items with large difficulty (minus 49) that shift to lower difficulties, increasing in 29 items for moderate difficulty, 16 in light difficulties and 4 entering into non-difficulty level. Together with Figure 13, it shows evidence of consistent overall improvement. Taking all data from Figure 14 and Equation (2) we obtain around a 14% mean improvement rate, being the mean value of all individual levels of restriction.

**Figure 15:**
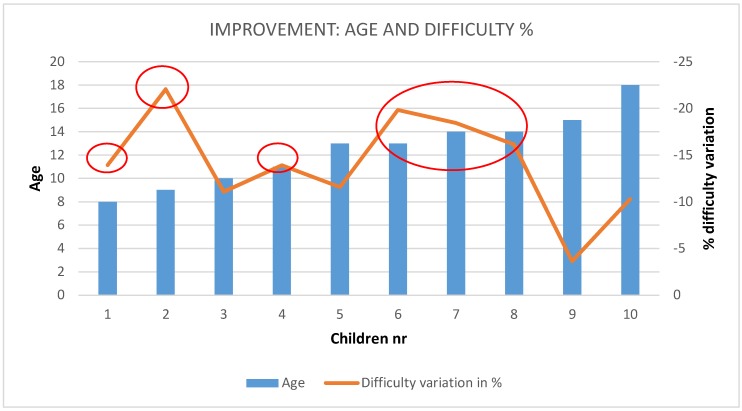
Age (blue, left axis) and improvement (% of decrease on degree of difficulty-right axis).

Figure 15 shows the improvement for each child, including age information in which no special correlation with age is found. Figure 15 red ellipses highlight meaningful improvements in the range of 10% to 22 % in more than half of the subjects.

### 4.4. Discussion

Data shows TOD may be a useful tool for training in time orientation capacities as agenda tasks.

All children participating in the experience show some improvement. This means the establishment of a conditioned support for the cause-effect relation, associating specific stimuli to corresponding tasks realization.

Improvement is measured in a neutral way regarding interpretation of mechanisms underlying it. It has an overall mean value around 14% decrease in restriction level for involved tasks.

A longer experiment is needed to conclude if changes are stable. Stable changes are considered learnings, whereas transitory ones are automatisms. Changes recorded so far refer mainly to the capacity of execution of a task or action more than to a learning process itself. Response then is mainly the establishment of a “basic conditioned reflex”, or cause-effect relation, as it is hearing the stimulus and triggering an action. In order to affect the synaptic connections involved, longer continued repetition of the process “stimulus-response” is needed. These findings though, evidence the possibility to have them learned.

Placement of the TOD in the classroom has been difficult in several scenarios. This was detected early in the experiment, so a manual to improve it was developed. Even so, difficulties remain, mainly because the excess of information hides the main information. In the visited special education classes there is plenty of visual material, posters, pictures, etc., which make it more difficult to focus attention to TOD messages.

## 5. Conclusions and Future Work

A design, development and evaluation of a Time Orientation Device for training and support has been completed. Several design cycles have reached a third prototype version which was adapted to special education goals and circumstances. It provides very large flexibility to configure notifications via a small screen, a luminous row of multicolor elements and associated sounds.

In addition to the development, an evaluation action has taken place, bringing up positive results. Trials in four special education schools have been performed. In order to evaluate it as an education tool a standard and world-wide classification has been used, the ICF from the WHO.

This communication describes the technology, methodology and logistics for such experiments. The action yielded positive results based in prior and after ICF assessments and observation register templates for teachers.

Improvements evidence potential benefit of the use of TOD, although they need to be considered as automatisms versus learnings, due to the length of the period involved.

Further evaluation is to be performed with specific tools to assess time orientation capacities in children. Our idea is to do so through examples and activities that are related with specific time cognitive functions, as sequencing or calculating duration. A good option we consider is to base them in KaTid method, for it was developed for that purpose and it has its roots in ICF classification of cognitive human functions related with time. Longer evaluation times will also allow conclusions regarding how deep into learning such improvements may go.

## Figures and Tables

**Table 1:** Participants per school and classes.

School	No. of Children	No. Classes	No. of Teachers and Collaborators
CPEE Alborada	6	6 Classes 1 Direction room	6 Teachers 3 Direction staff
San Martín de Porres (ATADES)	4	2 Classes	2 Teachers Director
CPEE Piaget	5	3 Classes	3 Teachers Studies coordinator
CPEE Rivière	1	1 Class 1 Staff room	1 Teacher Director
Total	16	Classes 12 Rooms 2 TODs 14	Teachers 12 Other staff 6

**Table 2:** Age of participants.

No.	Age
1	19
9	12 to 15
6	7 to 11

**Table 3:** Disability degrees of participants.

No.	% of Disability
3	mild disability from 25 to 49 %
12	severe disability from 50 to 95 %
1	not provided

**Table 4:** Curricular competence level of participants.

No. of Children	Curricular Competence Level
7	1st phase of child education (corresponding to 2 to 3 years old in normalized schools)
6	2nd phase of child education (corresponding to 4 to 5 years old in normalized schools)
3	With no registered information

**Table 5:** Difficulty percentage ranges per category and mean value taken for quantitative study.

Label	Level of Difficulty	Difficulty Range in %	Difficulty Mean Value (%)
0	no difficulty	0%	0%
1	light difficulty	5 to 24%	14.5%
2	moderate difficulty	25 to 49%	37%
3	large difficulty	50 to 95%	72.5%
4	total difficulty	96 to 100%	98%

**Table 6:** First evaluation data for child code 010201, 11 years old.

Code 010201 1st Assessment	No. of Items
No difficulty	0
light difficulty (5% a 24%)	0
moderate difficulty (25% a 49%)	4
large difficulty (50% a 95%)	12
total difficulty (96% a 100%)	0
ACTIVITIES WITH RESTRICTION	16
LEVEL OF RESTRICTION	63,63%

**Table 7:** Second evaluation data for child code 010201, 11 years old.

Code 010201 2nd Assessment	No. of Items
no difficulty	0
light difficulty (5% a 24%)	2
moderate difficulty (25% a 49%)	7
large difficulty (50% a 95%)	7
total difficulty (96% a 100%)	0
ACTIVITIES WITH RESTRICTION	16
LEVEL OF RESTRICTION	49.72%
